# Differential impact of body mass index and its change on the risk of breast cancer by molecular subtype: A case-control study in Japanese women

**DOI:** 10.1186/2193-1801-1-39

**Published:** 2012-10-23

**Authors:** Aiko Sueta, Hidemi Ito, Tania Islam, Satoyo Hosono, Miki Watanabe, Kaoru Hirose, Takashi Fujita, Yasushi Yatabe, Hiroji Iwata, Kazuo Tajima, Hideo Tanaka, Hirotaka Iwase, Keitaro Matsuo

**Affiliations:** 1 Division of Epidemiology and Prevention, Aichi Cancer Center Research Institute, 1-1, Kanokoden, Chikusa-ku, Nagoya, 464-8681 Japan; 2 Department of Breast and Endocrine Surgery, Kumamoto University Graduate School of Medical Science, 1-1-1, Honjo, Kumamoto, 860-8556 Japan; 3 Department of Planning and Information, Aichi Prefectural Institute of Public Health, 7-6, Azanagare, Tsujimachi, Kita-ku, Nagoya, 462-8576 Japan; 4 Department of Breast Oncology, Aichi Cancer Center Central Hospital, 1-1, Kanokoden, Chikusa-ku, Nagoya, 464-8681 Japan; 5 Department of Pathology and Molecular Diagnostics, Aichi Cancer Center Central Hospital, 1-1, Kanokoden, Chikusa-ku, Nagoya, 464-8681 Japan; 6 Aichi Cancer Center Research Institute, 1-1, Kanokoden, Chikusa-ku, Nagoya, 464-8681 Japan; 7 Department of Epidemiology, Nagoya University Graduate School of Medicine, 65, Tsurumai-cho, Showa-ku, Nagoya, 466-8550 Japan

**Keywords:** Body mass index, Breast cancer risk, Molecular subtype

## Abstract

Body mass index (BMI) is an independent risk factor for luminal-type breast cancer in Western populations. However, it is unclear whether the impact of BMI differs according to breast cancer subtype in Japanese populations. We conducted a case–control study with 715 cases and 1430 age- and menopausal status-matched controls to evaluate the associations of BMI and its change (from age 20 years to the current age) with breast cancer risk. We applied conditional logistic regression models to estimate odds ratios (ORs) and 95% confidence intervals (CIs). Tumor subtypes were divided into four subtypes, namely the luminal, luminal/HER2, HER2-rich, and triple-negative subtypes. Current BMI and BMI change were positively associated with postmenopausal breast cancer risk. On stratified analysis by tumor subtype, we observed associations between current BMI and BMI change and postmenopausal breast cancer risk for the luminal subtype, with OR for each 1 kg/m^2^ increase in current BMI of 1.14 (95% CI: 1.07 - 1.20) and the corresponding OR of BMI change of 1.16 (1.09 - 1.23) (each P_*trend*_ < 0.001). Additionally, we found the same tendency for the triple-negative subtype, with the OR for a 1 kg/m^2^ increase in current BMI of 1.21 (1.05 - 1.39) and that for BMI change of 1.18 (1.02 - 1.36) (P_*trend*_ was 0.008 and 0.024, respectively). In premenopausal women, a suggestive inverse association was observed between BMI change and breast cancer risk for the luminal subtype only, with OR of BMI change of 0.93 (0.87 - 1.00, *P*_trend_ = 0.054). No association was seen between BMI at age 20 years and risk of any tumor subtype. In conclusion, BMI and its change are associated with the risk of both luminal and triple-negative breast cancer among postmenopausal Japanese women. These findings suggest the etiological heterogeneity of breast cancer among tumor subtypes.

**Electronic supplementary material:**

The online version of this article (doi:10.1186/2193-1801-1-39) contains supplementary material, which is available to authorized users.

## Introduction

The rapid increase in the incidence rate of breast cancer over the last quarter of a century in Japan (Matsuda et al. 2011) can be related to changes in the prevalence of established risk factors, such as reproductive and anthropometric factors (Minami et al. 2004). Among these, obesity is an important and potentially modifiable risk factor for breast cancer, the incidence of which is increasing in most countries (World Health Organization 2011). In Japan also, body mass index (BMI) has consistently increased in older women, albeit that the prevalence of obesity remains lower than in Western countries (Funatogawa et al. 2009; OECD 2010).

The association between high BMI or adult weight gain and breast cancer risk for postmenopausal women has been established, and found to be partly attributable to an increase in circulating endogenous estrogen levels from adipose tissue, the primary source of estrogen in postmenopausal women (Han et al. 2006; Kawai et al. 2010; Eliassen et al. 2006). These findings are supported by the fact that the association between BMI and risk of postmenopausal breast cancer is stronger for estrogen receptor (ER)-positive than -negative tumors (Feigelson et al. 2006; Vrieling et al. 2010; Suzuki et al. 2011).

The clinical relevance of molecular subtypes of breast cancer has been demonstrated. These appear to include at least four major tumor subtypes defined by ER, progesterone receptor (PR), and human epidermal growth factor receptor 2 (HER2) status, (Perou et al. 2000; Sorlie et al. 2001). Although these distinct subtypes have been associated with different clinical outcomes (Sotiriou et al. 2003), evidence on etiologic differences among them is limited. Some epidemiological studies have shown that reproductive factors and BMI are associated with an increased risk of only ER- and/or PR-positive tumors (Phipps et al. 2011; Phipps et al. 2008), but others have suggested that ER- and PR-negative tumors are also positively associated with BMI (Yang et al. 2011; Yang et al. 2007). These studies were conducted mainly in Western populations, however, and the findings have not been entirely consistent. Additionally, potential biological differences among cancers may be present, and the prevalence of obesity differs between Western and Asian countries. These issues thus highlight the importance of evaluating the impact of BMI and its change on breast cancer risk by tumor subtype in Asian populations.

Here, we conducted a case–control study to evaluate associations between BMI and breast cancer risk by tumor subtype, defined by ER, PR, and HER2 status, in a Japanese population.

## Material and methods

### Subjects

Case subjects were 715 female cancer patients with no previous history of breast cancer who initially visited Aichi Cancer Center Hospital (ACCH) in Nagoya, Japan, between January 2001 and June 2005. Control subjects were 1430 age- and menopause-matched females without any history of cancer, giving a 1:2 case–control ratio. All subjects were selected from the database of the Hospital-based Epidemiologic Research Program at Aichi Cancer Center (HERPACC), the framework of which has been described elsewhere (Tajima et al. 2000; Hamajima et al. 2001). In brief, 23408 HERPACC-enrolled first-visit outpatients were asked to provide information on lifestyle factors and blood samples for genetic studies. Approximately 95% of eligible subjects completed a self-administered questionnaire, responses to which were checked by a trained interviewer, and 55% provided blood samples. We selected women as cases whose histological diagnosis was available and whose ER, PR and HER2 status results were confirmed. We used cancer-free patients at our hospital as controls given the likelihood that our case subjects arose within this population base. We previously showed that the general lifestyle of cancer-free outpatients at our hospital was in accord with that of a general population randomly selected from the electoral roll of Nagoya City, Aichi Prefecture, confirming the feasibility of their use as controls in epidemiological studies (Inoue et al. 1997). The present study was approved by the Institutional Ethics Review Board of ACC, and all participants gave written informed consent.

### Evaluation of environmental factors

The HERPACC questionnaire included items on height and weight, weight at age 20 years, menopausal status, parity and lactation, drinking and smoking habits, exercise, hormone use, individual medical history, family history of cancer, and referral pattern to our hospital. Patients were asked to provide information on their lifestyle at one year before the onset of symptoms for those who were symptomatic, or at the time of interview for those who were asymptomatic.

BMI at current age and at age 20 years was calculated as the weight divided by the squared height (kg/m^2^) and divided into four groups, < 18.5, ≥ 18.5 to < 22, ≥ 22 to < 25, and ≥ 25. BMI at current age was referred to BMI at cancer diagnosis. Although the cut-off points of BMI for underweight/normal/overweight/obese under the World Health Organization (WHO) definition are < 18.5, ≥ 18.5 to < 25, ≥ 25 to < 30, and ≥ 30, respectively (World Health Organization 2012), we combined the overweight and obese groups and divided the normal group into two subgroups in consideration of the low prevalence of obesity in Japan. BMI change from age 20 years to the current age was calculated as the difference between current BMI and BMI at age 20, and was also categorized into six groups, with a BMI loss of < −4, or ≥ −4 to < −2; stable BMI of ≥ −2 to < +2; and BMI gain of ≥ +2 to < +5, ≥ +5 to < +8, and ≥ +8. Alcohol consumption was divided into never, light, moderate, and heavy drinking, with light drinking defined as consumption of less than 5 g of ethanol per day; moderate drinking as between 5 and less than 23 g per day; and heavy drinking as 23 g or more per day. Smoking habit was divided into never, former or current smoking of < 20, and ≥ 20 pack-years. Regular exercise was classified into no exercise, less than half an hour of exercise per day, and half an hour or more of exercise per day. Hormone use included all use for contraception, infertility, treatment or hormone replacement therapy. Family history was considered positive when a mother or sister had breast cancer.

### Definition of tumor subtypes and their assessment

We performed a medical record review for all case subjects. ER, PR and HER2 expression was confirmed from the original pathological reports. ER and PR status was assessed according to the Allred system by combining frequency and intensity scores. Using the Allred score, tumors scoring 0 or 2 were regarded as negative, and 3 or more as positive. HER2 expression was determined by immunohistochemical (IHC) staining based on the Hercep test. Tumors that showed 2+ were definitively evaluated by fluorescence in situ hybridization (FISH). Tumors were considered HER2-positive if they were either scored 3+ by IHC staining, or 2+ by IHC and were also HER2 amplified on the basis of FISH. With regard to tumor subtypes, we adopted the IHC classification described by Carey et al. (Carey et al. 2006), which categorizes breast cancer according to the expression status of ER, PR and HER2 as follows: luminal (ER- and/or PR-positive and HER2-negative), luminal/HER2 (ER- and/or PR-positive and HER2-positive), HER2-rich (ER- and PR-negative and HER2-positive), and triple-negative (ER-, PR- and HER2-negative).

### Statistical analysis

The significance of differences in categorized demographic variables between cases and controls was evaluated using the Chi-square test. Subjects with unknown data were excluded from the Chi-square test. Mean ages were compared using the Mann–Whitney test. The effect of each BMI indicator (current BMI, BMI at age 20 years, and BMI change) was estimated using odds ratios (ORs) and 95% confidence intervals (CIs) by conditional logistic regression models adjusted for potential confounders, namely age, age at menarche (≤ 12, 13–14, ≥ 15), menopausal status (premenopause, postmenopause), parity (0, 1–2, ≥ 3), age at first live birth (nulliparous, 17–23, 24–27, ≥ 28), family history of breast cancer (yes, no), hormone use for contraception, infertility treatment or hormone replacement therapy (yes, no), total exercise (none, < 0.5 h/day, ≥ 0.5 h/day), and referral pattern to our hospital (patient discretion, recommendation by family or friend, referral from another clinic, secondary screening after primary screening, or other). We excluded 6 subjects (2 cases and 4 controls) with missing information on menopausal status, leaving 2139 subjects for stratified analysis by menopausal status.

Interactions between respective BMI and menopausal status were evaluated under the multiplicative model, and the cross-product terms of these factors were included in the multivariate conditional logistic regression models as interaction terms. The *P* value for interaction was calculated by a likelihood ratio test which compared models with and without the interaction terms.

All statistical analyses were carried out using STATA ver. 11 (Stata Corp, College Station, TX). All tests were two-sided and *P* values < 0.05 were considered statistically significant.

## Results

Table 1 shows the distribution of background characteristics of the 715 breast cancer cases and 1430 controls. Age and menopausal status were matched appropriately. Compared to control subjects, cases were more likely to have an early age at menarche (mean age ± SD: 13.3 ± 1.6 vs. 13.5 ± 1.7, *P* = 0.040), later first live birth among parous women (mean age ± SD: 26.2 ± 3.4 vs. 25.9 ± 3.6, *P* = 0.038), and a higher BMI (mean BMI ± SD: 22.5 ± 3.3 vs. 21.9 ± 3.2, *P* < 0.001). With regard to referral pattern, cases were more likely to have a family recommendation or referral from other clinics and less likely to refer at patient discretion or as part of secondary screening after primary screening. Unexpectedly, case subjects were more likely to do exercise for more than half an hour per day than controls (*P* = 0.029). In contrast, the other factors did not statistically significantly differ between the two groups.

**Table 1:** **Characteristics of breast cancer cases and cancer-free controls**

	Cases (n = 715) (%)	Controls (n = 1430) (%)	***P***
Age (year)			
≤29	10 (1.4)	25 (1.8)	
30-39	83 (11.6)	184 (12.9)	
40-49	195 (27.3)	387 (27.1)	
50-59	231 (32.3)	439 (30.7)	
60-69	147 (20.6)	295 (20.6)	
70-79	49 (6.9)	100 (7.0)	0.93
Mean age ± SD	52.4 ± 11.1	52.0 ± 11.3	0.410
Menopausal status			
Premenopausal	340 (47.6)	680 (47.6)	
Postmenopausal	373 (52.2)	746 (52.2)	1.00
Unknown	2 (0.3)	4 (0.3)	
Age at menarche(year)			
≤12	234 (32.7)	408 (28.5)	
13-14	336 (47.0)	670 (46.9)	
≥15	137 (19.2)	326 (22.8)	0.06
Unknown	8 (1.1)	26 (1.8)	
Mean age ± SD	13.3 ± 1.6	13.5 ± 1.7	0.040
Parity			
0	103 (14.4)	216 (15.1)	
1-2	462 (64.6)	895 (62.6)	
≥3	150 (21.0)	312 (21.8)	0.74
Unknown	0 (0)	7 (0.5)	
Age at first live birth (year)			
17-23	114 (15.9)	278 (19.4)	
24-27	302 (42.2)	614 (42.9)	
≥28	191 (26.7)	304 (21.3)	
No birth	103 (14.4)	216 (15.1)	0.02
Unknown	5 (0.7)	18 (1.3)	
Mean age ± SD	26.2 ± 3.4	25.9 ± 3.6	0.04
Age at menopause (year) (postmenopausal women only)			
≤49	128 (34.4)	247 (33.1)	
≥50	238 (63.8)	489 (65.6)	0.64
Unknown	7 (1.9)	10 (1.3)	
Mean age ± SD	49.6 ± 5.0	49.4 ± 5.1	0.71
BMI (kg/m^2^)			
<18.5	59 (8.3)	150 (10.5)	
18.5-21.9	293 (41.0)	679 (47.5)	
22–24.9	210 (29.4)	379 (26.5)	
≥25	148 (20.7)	210 (14.7)	< 0.001
Unknown	5 (0.7)	12 (0.8)	
Mean BMI ± SD	22.5 ± 3.3	21.9 ± 3.2	< 0.001
Alcohol consumption (g/day)			
Never	444 (62.1)	900 (62.9)	
Lighta	124 (17.3)	277 (19.4)	
Moderateb	84 (11.8)	166 (11.6)	
Heavyc	56 (7.8)	81 (5.7)	0.2
Unknown	7 (1.0)	6 (0.4)	
Smoking (pack/year)			
Never	594 (83.1)	1173 (82.0)	
<20	79 (11.1)	162 (11.3)	
≥20	34 (4.8)	84 (5.9)	0.560
Unknown	8 (1.1)	11 (0.8)	
Total exercise (hour/day)			
No	200 (28.0)	407 (28.5)	
<0.5	279 (39.0)	629 (44.0)	
≥0.5	228 (31.9)	384 (26.9)	0.03
Unknown	8 (1.1)	10 (0.7)	
Family history of breast cancer			
Yes	59 (8.3)	94 (6.6)	
No	656 (91.8)	1336 (93.4)	0.16
Hormone use			
Yes	98 (13.7)	197 (13.8)	
No	604 (84.5)	1189 (83.2)	0.88
Unknown	13 (1.8)	44 (3.1)	
Referral pattern to our hospital			
Patient's discretion	205 (28.7)	496 (34.7)	
Family recommendation	154 (21.5)	244 (17.1)	
Referral from other clinics	206 (28.8)	251 (17.6)	
Secondary screening after primary screening	139 (19.4)	431 (30.1)	
Others	6 (0.8)	4 (0.3)	< 0.001
Unknown	5 (0.7)	4 (0.3)	
Tumor subtype			
Luminal (ER and/or PR+ and HER2 -)	455 (63.6)		
Luminal HER2 (ER and/or PR+ and HER2+)	108 (15.1)		
HER2 rich (ER and PR- and HER2+)	84 (11.8)		
Triple negative (ER and PR- and HER2-)	68 (9.5)		

^a^Light drinker means consumption of < 5g ethanol/day. ^b^Moderate drinker means consumption of between 5 and 23 g ethanol/day. ^c^Heavy drinker means consumption of >23 g ethanol/day.

Table 2 shows the association between current BMI, BMI at age 20 years, or BMI change and breast cancer risk. For all women, we observed strong positive associations between current BMI or BMI change and breast cancer risk, with ORs in the overweight group (current BMI ≥ 25) of 1.61 (95% CI : 1.23 - 2.11) compared to the normal group (BMI range from 18.5 to 22) (*P*_trend_ < 0.001). Similarly, OR for women with the highest BMI change (BMI gain ≥ 8) was 2.56 (1.49 - 4.38) compared to those of women with stable BMI change (BMI change from −2 to +1.9) (*P*_trend_ < 0.001). In contrast, no significant association was seen between BMI at age 20 years and breast cancer risk. On stratified analysis by menopausal status, current BMI and BMI change were positively associated with breast cancer risk only among postmenopausal women (each *P*_trend_ < 0.001). We also observed statistically significant interactions between current BMI, BMI change, and menopausal status (*P*_interaction_ = 0.0036 and *P*_interaction_ < 0.001, respectively).

**Table 2:** **Odds ratios (ORs) of breast cancer by the distribution of body mass index (BMI)**

		All (n=2145)			Premenopausal (n=1020)			Postmenopausal (n=1119)		
	Case / Control			Case / Control			Case / Control			
	(n=715 / 1430)	ORs	95% CI	(n=340 / 680)	ORs	95% CI	(n=373 / 746)	ORs	95% CI	***P***_interaction_
Current BMI (kg / m^2^)										
< 18.5	59 / 150	0.93	0.66 - 1.33	41 / 83	1.11	0.70 - 1.76	17 / 67	0.66	0.36 - 1.23	
18.5 - 21.9	293 / 679	1.00	(ref.)	175 / 377	1.00	(ref.)	118 / 299	1.00	(ref.)	
22–24.9	210 / 379	1.26	0.99 - 1.59	84 / 144	1.08	0.75 - 1.55	125 / 234	1.38	0.99 - 1.93	
≥ 25	148 / 210	1.61	0.99 - 1.59	36 / 74	0.88	0.55 - 1.41	112 / 136	1.98	1.39 - 2.82	
Unknown	5 / 12			4 / 2			1 / 10			
per1 kg / m^2^ increase		1.06	1.02 - 1.09		0.98	0.93 - 1.03		1.11	1.06 - 1.15	
*Ptrend*			< 0.001			0.373			< 0.001	0
BMI at age 20 years										
< 18.5	120 / 295	0.79	0.61 - 1.01	72 / 174	0.81	0.57 - 1.14	47 / 119	0.74	0.50 - 1.09	
18.5 - 21.9	452 / 842	1.00	(ref.)	221 / 412	1.00	(ref.)	230 / 429	1.00	(ref.)	
22–24.9	116 / 229	0.91	0.70 - 1.19	39 / 75	0.88	0.55 - 1.39	77 / 153	0.95	0.67 - 1.33	
≥ 25	17 / 35	0.81	0.43 - 1.51	5 / 12	0.77	0.25 - 2.39	12 / 23	0.88	0.40 - 1.92	
Unknown	10 / 29			3 / 7			7 / 22			
per1 kg / m^2^ increase		1.02	0.98 - 1.07		1.03	0.96 - 1.11		1.02	0.96 - 1.08	
*Ptrend*			0.346			0.359			0.601	0.88
BMI change (from age 20 to the current age)										
< −4	9 / 24	0.72	0.31 - 1.65	5 / 5	1.88	0.49 - 7.19	4 / 19	0.40	0.12 - 1.33	
-4 to −2.1	30 / 83	0.80	0.51 - 1.26	16 / 27	1.22	0.62 - 2.42	14 / 56	0.58	0.30 - 1.14	
-2 to +1.9	335 / 717	1.00	(ref.)	199 / 402	1.00	(ref.)	135 / 314	1.00	(ref.)	
+2 to +4.9	221 / 421	1.05	0.84 - 1.32	86 / 185	0.86	0.61 - 1.22	134 / 233	1.15	0.83 - 1.58	
+5 to +7.9	72 / 122	1.30	0.92 - 1.86	20 / 39	1.07	0.56 - 2.05	52 / 83	1.42	0.91 - 2.20	
≥ +8	34 / 28	2.56	1.49 - 4.38	8 / 15	0.96	0.37 - 2.46	26 / 13	4.42	2.10 - 9.28	
Unknown	14 / 35			6 / 7			8 / 28			
per1 kg / m^2^ increase		1.06	1.03 - 1.10		0.95	0.90 - 1.01		1.12	1.07 - 1.17	
*Ptrend*			< 0.001			0.102			< 0.001	< 0.001

ORs were adjusted for age, age at menarche, menopause, parity, age at first live birth, family history of breast cancer, total exercise, hormone use, and referral pattern to our hospital.

^a^Analysis of the likelihood test for equality between BMI and menopausal status.

Furthermore, we also evaluated the association between BMI and breast cancer risk by tumor subtype (Table 3). As the effect of BMI by menopausal status showed heterogeneity, we obtained stratified results by menopausal status. Current BMI and BMI change were positively associated with breast cancer risk for both luminal and triple-negative subtypes among postmenopausal women only, with ORs for each 1 kg/m^2^ increase in current BMI of 1.14 (1.07 - 1.20) for the luminal subtype (*P*_trend_ < 0.001) and 1.21 (1.05 - 1.39, *P*_trend_ = 0.008) for the triple-negative subtype. Similarly, the effect of BMI change was statistically significant for the luminal and triple-negative subtypes among postmenopausal women (*P*_trend_ < 0.001 and *P*_trend_ = 0.024, respectively). Further, we also found the same tendency for increased risk with BMI change after adjustment for BMI at age 20 years (data was not shown). No significant associations between current BMI, BMI change and breast cancer risk were seen for the other subtypes.

**Table 3:** **Associations between BMI and breast cancer risk by tumor subtype and menopausal status**

	Luminal (n=455)						Luminal / HER2 (n=108)						HER2 (n=84)						Triple negative (n=68)					
	Premenopausal			Postmenopausal			Premenopausal			Postmenopausal			Premenopausal			Postmenopausal			Premenopausal			Postmenopausal		
	Case			Case			Case			Case			Case			Case			Case			Case		
	(n=236)	ORs	95% CI	(n=218)	ORs	95% CI	(n=56)	ORs	95% CI	(n=52)	ORs	95% CI	(n=29)	ORs	95% CI	(n=55)	ORs	95% CI	(n=19)	ORs	95% CI	(n=48)	ORs	95% CI
Current BMI (kg / m^2^)																								
< 18.5	30	1.23	0.70 - 2.15	9	0.65	0.28 - 1.50	5	0.63	0.16 - 2.56	2	0.66	0.09 - 4.83	3	0.48	0.04 - 5.64	4	0.34	0.06 - 1.82	3	-		2	0.43	0.04 - 4.12
18.5 - 21.9	123	1.00	(ref.)	61	1.00	(ref.)	31	1.00	(ref.)	23	1.00	(ref.)	10	1.00	(ref.)	22	1.00	(ref.)	11	1.00	(ref.)	12	1.00	(ref.)
22–24.9	51	0.92	0.59 - 1.45	76	1.46	0.94 - 2.29	17	1.95	0.64 - 5.99	13	0.81	0.27 - 2.42	12	3.67	0.69 - 19.5	18	0.80	0.26 - 2.47	4	-		18	2.74	0.81 - 9.22
≥ 25	28	0.96	0.55 - 1.68	71	2.13	1.33 - 3.42	3	0.17	0.03 - 1.03	14	2.38	0.75 - 7.58	4	2.66	0.13 - 52.8	11	1.04	0.33 - 3.30	1	-		16	7.51	1.84 - 9.22
Unknown	4			1			0			0			0			0			0			0		
per1 kg / m^2^ increase		0.97	0.92 - 1.03		1.14	1.07 - 1.20		0.96	0.81 - 1.13		1.14	0.99 - 1.30		1.18	0.91 - 1.53		1.05	0.93 - 1.18		-			1.21	1.05 - 1.39
*Ptrend*			0.363			<0.001			0.620			0.071			0.222			0.452						0.008
BMI at age 20 years																								
< 18.5	45	0.72	0.46 - 1.11	29	0.81	0.48 - 1.36	10	0.36	0.12 - 1.10	7	0.53	0.17 - 1.65	11	4.32	0.63 - 29.7	8	0.75	0.22 - 2.55	6	-		3	0.57	0.13 - 2.50
18.5 - 21.9	154	1.00	(ref.)	127	1.00	(ref.)	41	1.00	(ref.)	34	1.00	(ref.)	15	1.00	(ref.)	35	1.00	(ref.)	11	1.00	(ref.)	34	1.00	(ref.)
22 - 24.9	33	0.98	0.58 - 1.64	51	0.98	0.63 - 1.51	2	0.08	0.01 -0.56	9	1.71	0.40 - 7.37	3	20.9	1.03 - 421.7	9	2.23	0.63 - 7.91	1	-		8	0.79	0.26 - 2.35
≥ 25	2	0.40	0.07 - 2.16	6	0.97	0.32 - 3.00	2	0.58	0.01 - 23.7	2	1.10	0.07 - 17.5	0	-		2	2.99	0.17 - 53.9	1	-		2	0.54	0.06 - 4.96
Unknown	2			5			1			0			0			1			0			1		
per1 kg / m^2^ increase		1.06	0.97 - 1.15		1.02	0.94 - 1.11		0.98	0.79 - 1.23		1.13	0.89 - 1.42		1.04	0.90 - 2.04		1.13	0.91 - 1.41		-				0.98
*Ptrend*			0.235			0.617			0.876			0.314			0.852			0.264						0.838
BMI change (from age 20 to the current age)																								
< −4	3	1.27	0.25 - 6.38	4	0.81	0.21 - 3.16	1	0.47	0.01 - 19.5	0	-		0	-		0	-		1	-		0	-	
-4 to −2.1	14	1.18	0.56 - 2.48	8	0.71	0.30 - 1.70	2	1.09	0.14 - 8.64	0	-		0	-		4	0.74	0.06 - 8.53	0	-		2	0.62	0.08 - 4.87
−2 to +1.9	141	1.00	(ref.)	64	1.00	(ref.)	32	1.00	(ref.)	26	1.00	(ref)	15	1.00	(ref.)	27	1.00	(ref.)	11	1.00	(ref.)	18	1.00	(ref.)
+2 to +4.9	58	0.79	0.52 - 1.20	84	1.41	0.91 - 2.18	17	1.02	0.37 - 2.81	18	0.89	0.33 - 2.39	6	0.60	0.07 - 5.25	13	0.34	0.11 - 0.99	5	-		19	2.33	0.70 - 7.76
+5 to +7.9	9	0.55	0.22 - 1.38	34	1.90	1.05 - 3.41	3	1.05	0.17 - 6.54	5	0.44	0.10 - 1.91	6	12.1	0.75 - 195.9	8	0.85	0.21 - 3.36	2	-		5	3.13	0.45 - 21.7
≥ +8	6	0.98	0.33 - 2.96	18	5.50	2.14 - 14.1	0	-		3	5.66	0.31 - 102.3	2	-		2	2.40	0.17 - 33.3	0	-		3	4.41	0.31 - 61.9
Unknown	5			6			1			0			0			1			0			1		
per1 kg / m^2^ increase		0.93	0.87 - 1.00		1.16	1.09 - 1.23		0.96	0.80 - 1.16		1.10	0.95 - 1.27		1.35	0.94 - 1.95		1.00	0.88 - 1.14					1.18	1.02 - 1.36
*Ptrend*			0.054			<0.001			0.670			0.197			0.108			0.982						0.024

ORs were adjusted for age, age at menarche, menopause, parity, age at first live birth, family history of breast cancer, total exercise, hormone use, and referral pattern to our hospital.

In premenopausal women, the suggestive inverse association of BMI change and breast cancer risk was observed only for the luminal subtype, with an OR for each 1 kg/m^2^ increase in BMI change of 0.93 (95% CI = 0.87 - 1.00, *P*_trend_ = 0.054). We were unable to assess ORs adequately among premenopausal women, particularly for the triple-negative subtype, because of the paucity of subjects. To guess the associations among them, we also showed an overall impact of BMI on risk (Additional file 1: Table S1). The results were similar to those when only postmenopausal women were included, but the magnitude of association of current BMI and BMI change with risk of the triple-negative subtype was greater among postmenopausal women only than in all women. This might suggest there were no trends of an increased risk for the triple negative subtype among premenopausal women.

We observed no association between BMI at age 20 years and risk for any tumor subtype.

## Discussion

In this study, we evaluated the associations between current BMI, BMI at age 20 years, BMI change and breast cancer risk. We showed the possibility that heterogeneity may be present in the association between adult weight gain and breast cancer risk by tumor subtype. Postmenopausal women with luminal and triple-negative breast cancers showed the same impact of BMI on breast cancer risk, despite the molecular and clinical differences between these two subtypes. These findings are consistent with those of several (Phipps et al. 2011; Phipps et al. 2008) but not all previous studies (Yang et al. 2007; Yang et al. 2011; Tamimi et al. 2011).

The biological mechanisms underlying these associations are unclear, but it is assumed that obesity influences breast cancer risk through multiple mechanisms. One is a classic estrogen-dependent mechanism, in which obesity contributes to lower serum levels of sex hormone-binding globulin and higher circulating levels of endogenous estrogen (Potischman et al. 1996). Almost all reproductive factors that influence estrogen levels are primarily associated with luminal-type tumors, but there is also some evidence related to risk for non-luminal-type tumors (Yang et al. 2007; Yang et al. 2011; Tamimi et al. 2011; Islam et al. 2012). For example, our recent study in Japanese suggested that age at menarche was inversely associated with the risk of both luminal and triple-negative breast cancer (Islam et al. 2012). Similarly, the Polish Breast Cancer Study showed an association of earlier age at menarche with basal-like tumors only (Yang et al. 2007). Other mechanisms might relate to non-hormonal factors, such as an insulin-related mechanism involving insulin, insulin resistance and insulin-like growth factors (IGFs), or an inflammation-related mechanism (Stoll 2002; Kern et al. 2001). IGFs, which stimulate mitogen-activated protein kinase (MAPK) or Akt and increase cell survival, proliferation, and migration through signaling cascades, are also considered to be risk factors for breast cancer (Hankinson et al. 1998; Schernhammer et al. 2005). Davison et al. (Davison et al. 2011) reported that IGFs promote cell survival in triple-negative breast cancer cells. Further, Maiti et al. (Maiti et al. 2010) suggested that metabolic syndrome, characterized by high BMI and insulin resistance, is significantly more prevalent in triple-negative than non-triple-negative patients. On the basis of these findings, we hypothesize that the combination of these complex mechanisms contributes to an increased risk of breast cancer, particularly of triple-negative subtypes. An understanding of the etiology of less common subtypes such as triple-negative tumors, which are the most clinically aggressive, may be useful in the development of prevention strategies.

Our observation of an inverse association between BMI gain and the risk of premenopausal luminal breast cancer is to our knowledge the first description in an Asian population. A number of studies have evaluate the effect of weight change on the risk of premenopausal breast cancer, but most evidence to date is from studies of ER and PR rather than HER2 disease, and the results are inconsistent (Huang et al. 1997; Michels et al. 2012; John et al. 2010; Weiderpass et al. 2004; Lahmann et al. 2005). In the Nurses’ Health Study, weight loss since age 18 years was inversely related to both ER+/PR+ and ER-/PR- tumors (Michels et al. 2012). In contrast, a case–control study in the San Francisco Bay Area reported that inverse associations between obesity and premenopausal breast cancer risk were limited to ER+/PR+ tumors (John et al. 2010), which is consistent with our present findings. The mechanisms underlying this inverse association remain to be elucidated. Several studies have suggested that women who are obese prior to menopause are likely to have ovulatory insufficiency or anovulatory cycles, resulting in decreased estrogen and progesterone levels (Suzuki et al. 2011; Baer et al. 2010; Kawai et al. 2010; Key & Pike 1988), and we accept this theory in interpreting our present result. However, given our limited number of subgroup subjects and the inconsistent results across studies, additional studies with sufficient numbers of subjects of various ethnicities are needed.

With regard to BMI at age 20 years, we found no statistically significant association, but rather a slightly inverse association when underweight women (BMI < 18.5) were excluded (Table 2). Several studies have suggested an inverse association between body weight in early adulthood and the risk of breast cancer (Suzuki et al. 2011; Baer et al. 2010; Kawai et al. 2010), but others have found no significant association (Sanderson et al. 2002; Okasha et al. 2003). A conclusive answer to this issue awaits further study.

Our study had several methodological strengths. First, it was conducted within the framework of the HERPACC study, which has a substantial number of subjects and a high response rate to completion of the questionnaire (95%). Second, the potential confounding factors of age and menopausal status were adjusted for by matching. Finally, the single-institution design of the study obviated the possibility of hormonal receptor or HER2 status misclassification resulting from different assay methods.

Since the present study was based on a hospital-based case–control study, several methodological limitations exist. First, the values for self-reported life-style factors considered as potential confounding factors might have been inaccurate. In particular, information about body weight at age 20 years was collected retrospectively. Second, the classification of tumor subtypes as defined in our study is not identical to other published data due to the lack of information on other tumor markers, such as cytokeratin (CK) 5/6 expression, epidermal growth factor receptor (EGFR), and Ki67. We were therefore unable to precisely distinguish between basal-like and triple-negative tumors, or between luminal A and luminal B tumors. Finally, subject numbers in our subgroup analyses were limited.

In conclusion, we found that current BMI and adult weight gain are associated with the risk of luminal and triple-negative breast cancer among postmenopausal women only. Our results provide additional evidence for these associations and suggest further etiological heterogeneity among tumor subtypes.

## Electronic supplementary material

Additional file 1: **Table S1.** Associations between BMI and breast cancer risk by tumor. (XLS 40 KB)

## Abbreviations

BMI: Body mass index
OR: Odds ratio
95%CI: 95% confidence interval
ER: Estrogen receptor
PR: Progesterone receptor
HER2: Human epidermal growth factor receptor 2
ACCH: Aichi Cancer Center Hospital
HERPACC: Hospital-based epidemiologic research program at aichi cancer center
WHO: World health organization
IHC: Immunohistochemistry
FISH: Fluorescence in situ hybridization
IGF: Insulin growth factor
CK: Cytokeratin
EGFR: Epidermal growth factor receptor.
